# Poisson balanced spiking networks

**DOI:** 10.1371/journal.pcbi.1008261

**Published:** 2020-11-20

**Authors:** Camille E. Rullán Buxó, Jonathan W. Pillow

**Affiliations:** 1 Center for Neural Science, New York University, New York City, New York, USA; 2 Princeton Neuroscience Institute, Princeton University, Princeton, New Jersey, USA; Ghent University, BELGIUM

## Abstract

An important problem in computational neuroscience is to understand how networks of spiking neurons can carry out various computations underlying behavior. Balanced spiking networks (BSNs) provide a powerful framework for implementing arbitrary linear dynamical systems in networks of integrate-and-fire neurons. However, the classic BSN model requires near-instantaneous transmission of spikes between neurons, which is biologically implausible. Introducing realistic synaptic delays leads to an pathological regime known as “ping-ponging”, in which different populations spike maximally in alternating time bins, causing network output to overshoot the target solution. Here we document this phenomenon and provide a novel solution: we show that a network can have realistic synaptic delays while maintaining accuracy and stability if neurons are endowed with conditionally Poisson firing. Formally, we propose two alternate formulations of Poisson balanced spiking networks: (1) a “local” framework, which replaces the hard integrate-and-fire spiking rule within each neuron by a “soft” threshold function, such that firing probability grows as a smooth nonlinear function of membrane potential; and (2) a “population” framework, which reformulates the BSN objective function in terms of expected spike counts over the entire population. We show that both approaches offer improved robustness, allowing for accurate implementation of network dynamics with realistic synaptic delays between neurons. Both Poisson frameworks preserve the coding accuracy and robustness to neuron loss of the original model and, moreover, produce positive correlations between similarly tuned neurons, a feature of real neural populations that is not found in the deterministic BSN. This work unifies balanced spiking networks with Poisson generalized linear models and suggests several promising avenues for future research.

## Author summary

A central idea in neuroscience is that populations of neurons work together to efficiently perform computations, although just how they do that remains unclear. Boerlin *et al* (2013) proposed a powerful framework for embedding linear dynamical systems into populations of spiking neurons, which they called balanced spiking networks (BSNs). Their approach starts from the principle that neurons greedily fire spikes to reduce error in the network output. Here we focus on a key limitation of this framework, which is that the network may become unbalanced in the presence of physiologically plausible communication delays. To overcome this shortcoming, propose two different extensions of the BSN framework that rely on probabilistic spiking. In our first model, we replace deterministic spiking of the original BSN with a Poisson spiking rule. In the second, we re-formulate the BSN objective so that Poisson spiking emerges as a way to reduce the expected network error. Our work brings the BSN framework closer to biological realism by increasing the stability and, most importantly, allowing communication delays between neurons without sacrificing accuracy. Furthermore, both probabilistic approaches reproduce key experimentally observed spiking behaviors of neural populations.

## Introduction

The brain carries out a wide variety of computations that can be implemented by dynamical systems, from sensory integration [1–4], to working memory [5–7], to movement planning and execution [8–10]. Although the existence of such computations in the brain is well established, the mechanisms by which these computations are implemented in networks of neurons remains poorly understood. One approach to this problem involves statistical modeling, which uses descriptive statistical methods to infer the dynamics of neural activity from recorded spike trains [10–21]. A second approach involves theoretical modeling, which seeks to identify strategies for implementing dynamical systems with networks of idealized model neurons [2, 22–30]. An important example of this second approach is the *balanced spiking network* (BSN) framework introduced by Boerlin *et al* [31].

The BSN model consists of a network of coupled leaky integrate-and-fire (LIF) neurons that can emulate an arbitrary linear dynamical system (LDS). The motivating idea is to design a network that approximates the output of a target LDS with a weighted combination of filtered spike trains. The population is divided into “excitatory” and “inhibitory” populations of neurons, based on whether they contribute positively or negatively to the output. This leads to an intuitive spiking rule: a neuron should spike whenever doing so will reduce the error between the output of the target LDS and the network output, i.e., the weighted combination of filtered spikes emitted so far. To make this work, each neuron has to maintain an internal representation of the error between the desired LDS output and the current network output. Boerlin *et al* showed that, remarkably, this computation can be mapped precisely onto the dynamics of an LIF neuron. A neuron’s membrane potential is a local representation of the network-wide error between target output and current network output, and its spike threshold is proportional to the amount by which adding a spike will reduce this error.

The BSN framework has many appealing characteristics. Spiking is efficient, in the sense that every spike contributes meaningfully to reducing error between target and actual output. The computations performed by the model are robust to perturbations and to the loss of neurons. The model also generates irregular spiking activity with intervals that that resemble those observed in real neurons.

However, the original BSN model has an important shortcoming that limits its plausibility as a model for information processing in real neural circuits. Namely, the model requires unrealistically fast propagation of information between neurons. Because every neuron’s membrane potential is tracking the overall error between target and actual output, the membrane potential of *all* neurons has to reset whenever any neuron emits a spike. Failure to impose this reset leads to increased activity as multiple neurons attempt to correct same error. In fact, implementations of the BSN model typically impose a rule enforcing that only one neuron is allowed to spike and required to immediately reset in a single time bin, effectively allowing spikes to propagate faster than the temporal resolution of the simulation (e.g., 0.1ms). Without this rule, the network can easily enter unstable modes in which excitatory and inhibitory populations emit massive spike bursts in alternating time bins, overshooting the target in an attempt to correct the error from the previous time bin.

Here we show that a probabilistic spiking rule can overcome the need for unrealistically fast propagation of spikes in the BSN framework. The basic intuition for our solution is that instead of making neurons spike deterministically whenever doing so will reduce error, we can allow multiple neurons to spike probabilistically such that error will be reduced on average.

We propose two alternate formulations of BSN with Poisson spiking, distinguished from each other by the level at which the network is attempting to minimize the decoding error. First, we describe a ‘local’ framework, which preserves the original BSN model dynamics but replaces the hard integrate-and-fire spiking rule with the soft firing threshold of the Poisson generalized linear model (GLM) [32–35]. This spiking rule generates stochastic spiking conditioned only on each neuron’s local copy of the error which, on average, leads to a reduction in the population-level read-out error.

Second, we propose a ‘population’ framework that replaces the greedy, single-neuron perspective of the local and BSN models with a rule based on minimizing the expected error at the population level. A vector of spike rates is generated by calculating the expected spike counts that minimizes the total decoding error. The probability of a single neuron spiking depends on its own weight, as with the local rule, but also takes into account the activity of the entire population of neurons and their weights. This coordination leads to spiking activity that is efficient and invariant to network size. Then, we show that both the local- and population-level Poisson frameworks make the BSN robust to synaptic delays. Finally, we compare all three frameworks and show that our local and population frameworks preserve the coding accuracy and robustness to neuron loss of the original BSN, while displaying more biologically plausible correlations between similarly tuned neurons.

This paper is organized as follows. We begin with a pedagogical review to the BSN model (Sec. 2). We then examine the model’s dependence on instantaneous spike propagation, and document the unstable behavior that arises if multiple spikes are allowed in a single time bin (Sec. 3). To address this shortcoming, we introduce local and population BSN models with conditionally Poisson spiking (Sec. 4). We go on to illustrate the accuracy and robustness of these models to synaptic transmission delays (Sec. 5). Finally, we compare the network performance of these models to that of the original BSN (Sec. 6).

## Results

### Background: Balanced spiking network model

**Fig 1:**
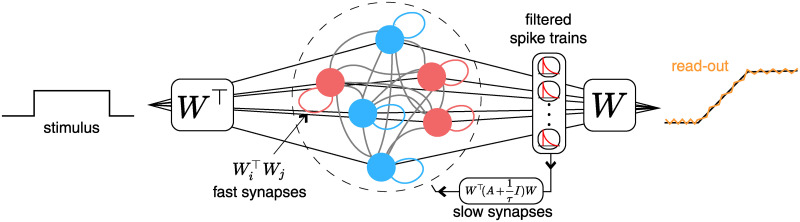
A diagram illustrating the BSN. Neurons receive stimulus input projected onto the transpose of a set of linear weights, *W*^⊤^, and the output is reconstructed by filtering spikes through the same weights, *W*. Neurons are connected via two coupling weights: fast synapses, *W*^⊤^*W*, which instantaneously propagate individual spikes through the network, and slow synapses, $W{}^{⊤}\left(A+\frac{1}{\tau }\right)W$, which implement network dynamics by feeding the filtered spike trains back into all neurons in the network. The network is divided into two equal populations of positive (red) and negative (blue) output weights, whose spikes have opposite effects on network output. Self-connections for these neurons are shaded in their respective colors, for visualization, but are always negative.

Here we provide a brief introduction to the original balanced spiking network (BSN) framework introduced by Boerlin, Machens, & Denève [31]. The goal is to design a spiking network that can accurately implement an arbitrary linear dynamical system. Consider a linear dynamical system defined by:

$$\begin{array}{c} \dot{x}\left(t\right)=Ax\left(t\right)+c\left(t\right),\;\left(\text{target}\;\text{dynamics}\right) \end{array}$$ (1)

where **x**(*t*) = (*x*_1_(*t*)…*x*_*J*_(*t*))^⊤^ is a vector of *J* dynamic variables that we will refer to as the *target*, *A* is the *J* × *J* linear dynamics matrix, and **c**_*t*_ = (*c*_*i*_(*t*), …*c*_*J*_(*t*))^⊤^ is a *J*-dimensional vector of inputs. The BSN model consists of a spiking network of *N* neurons that attempts to approximate the target output **x**(*t*) via a weighted combination of filtered spike trains:

$$\begin{array}{c} \hat{x}\left(t\right)=Wr\left(t\right)\;\left(\text{network}\;\text{readout}\right) \end{array}$$ (2)

where **r**(*t*) is the set of spike trains convolved with an exponential decay function, and *W* are *J* × *N* readout weights. (See Fig 1 for a schematic.) In general, for a 1-D dynamical system, the population is divided into equal pools of ‘positive’ and ‘negative’ neurons (depending on the signs of their individual weight components) although this is not a strict requirement. For *J* > 1, the ‘positive’ vs ‘negative’ distinction does not necessarily apply as the signs of the weights need not be consistent across dimensions.

The *i*’th component of the vector **r**(*t*) is given by

$$\begin{array}{c} r_{i}\left(t\right)=s_{i}\left(t\right)*h\left(t\right)=\int_{0}^{t}e^{-t^{\prime }/\tau }\;s_{i}\left(t^{\prime }\right)dt^{\prime },\;\left(\text{filtered}\;\text{spike}\;\text{trains}\right) \end{array}$$ (3)

where $s_{i}\left(t\right)=\sum_{t_{sp}^{i}}\delta \left(t-t_{sp}^{i}\right)$ denotes the *i*’th neuron’s spike train, defined by a series of delta functions at spike times $\left\{t_{sp}^{i}\right\}$, and *τ* is the time constant of the exponential filter *h*(*t*).

From this starting assumption, Boerlin *et al* introduce a greedy update rule that causes a neuron to spike whenever doing so will reduce the squared error between target **x**(*t*) and network output $\hat{x}\left(t\right)$,

$$\begin{array}{c} E\left(t\right)=||x\left(t\right)-\hat{x}\left(t\right){\left|\right|}_{2}^{2},\;\left(\text{error}\;\text{function}\right) \end{array}$$ (4)

which is mathematically equivalent to the threshold-crossing spiking rule in a leaky integrate-and-fire (LIF) neuron.

Here we recapitulate the derivation of this spiking rule in discrete time, for clarity and ease of implementation. Let ${\hat{x}}_{t}=Wr_{t}$ denote the network output at time bin *t*. The effect of adding a spike from neuron *i* in this time bin would be to augment the output vector by that neuron’s decoding weight vector **w**_*i*_, which is given by *i*’th row of the decoding weight matrix *W*. Thus, network output is ${\hat{x}}_{t}$ if neuron *i* is silent and ${\hat{x}}_{t}+w_{i}$ if it spikes. This suggests that the neuron should spike if doing so will result in smaller error, or

$$\begin{array}{c} \left|\right|x_{t}-\left({\hat{x}}_{t}+w_{i}\right){\left|\right|}_{2}^{2}<||x_{t}-{\hat{x}}_{t}{\left|\right|}_{2}^{2}. \end{array}$$ (5)

Simplifying this expression yields the condition that the neuron should spike if projection of the error vector onto **w**_*i*_ is greater than half the squared *L*_2_ norm of **w**_*i*_:

$$\begin{array}{c} w_{i}{}^{⊤}\left(x_{t}-{\hat{x}}_{t}\right)>\frac{1}{2}\left|\right|w_{i}{\left|\right|}_{2}^{2}.\;\left(\text{spike}\;\text{condition}\;\text{for}\;\text{neuron}\;i\right) \end{array}$$ (6)

Boerlin *et al* therefore suggest regarding the time-dependent left hand side of (Eq 6) as the membrane potential for neuron *i*, and the right hand side as its spike threshold *T*_*i*_:

$$\begin{array}{cccc} v_{[i]t} & =w_{i}{}^{⊤}\left(x_{t}-{\hat{x}}_{t}\right)\; & & \left(\text{membrane}\;\text{potential}\right) \end{array}$$ (7)

$$\begin{array}{cccc} T_{i} & =\frac{1}{2}\left|\right|w_{i}{\left|\right|}_{2}^{2} & & (\text{threshold}). \end{array}$$ (8)

Under this view, each neuron is computing a local approximation to the difference between the true target **x**_*t*_ and the network’s current output ${\hat{x}}_{t}=Wr_{t}$, projected onto that neuron’s weight vector **w**_*i*_.

The only missing piece from this expression is that the neurons do not of course have access to the true value of **x**_*t*_. But they do have implicit access to *A*, and thus to the dynamics governing **x**_*t*_. Boerlin *et al* therefore propose that the the network output ${\hat{x}}_{t}$ is sufficiently close to the target output **x**_*t*_ that it can be used to accurately approximate the desired dynamics: $Ax_{t}\approx A{\hat{x}}_{t}$. To make this explicit, we introduce a proxy variable **z**_*t*_, which denotes the network’s own (internal) approximation to the true target **x**_*t*_. This proxy variable evolves according to

$$\begin{array}{cc} z_{t} & =z_{t-1}+\Delta \left(A{\hat{x}}_{t-1}+c_{t}\right)\\ & =z_{t-1}+\Delta \left(AWr_{t-1}+c_{t}\right),\;\left(\text{proxy}\;\text{variable}\right) \end{array}$$ (9)

where for simplicity we use a forward Euler method for integrating the dynamics equation (Eq 1) with time bin size Δ. Higher accuracy can be achieved using exponential Euler integration (see Methods). Of course **z**_*t*_ is never represented explicitly; the network tracks **z**_*t*_ via its projection onto the decoding weights *W*, as we will see shortly.

#### Simulating the BSN model

Simulating the BSN model for a single time bin can be described by a sequence of three steps:

1. Calculate the “pre-spike” membrane potential for each neuron by combining inputs from the previous time step and external input.
2. Apply the threshold to determine which neurons (if any) emit spikes.
3. Reset to obtain “post-spike” membrane potentials **v**_*t*_ and update filtered spike trains **r**_*t*_.

We will describe each of these steps in turn. First, the update rule for the pre-spike membrane potential (consistent with Eq 7) is:

$$\begin{array}{cc} v_{[i]t}^{\left(pre\right)} & =w_{i}{}^{⊤}\left(z_{t}-{\hat{x}}_{t}^{\left(pre\right)}\right)\\ & =w_{i}{}^{⊤}(z_{t-1}+\Delta \left(AWr_{t-1}+c_{t}\right)-\left(1-\Delta \frac{1}{\tau }\right)Wr_{t-1})\\ & =v_{[i]t-1}+\Delta w_{i}{}^{⊤}(\left(A+\frac{1}{\tau }I\right)Wr_{t-1}+c_{t})\;\left(\text{pre}-\text{spike}\;\text{membrane}\;\text{potential}\right) \end{array}$$ (10)

where $v_{[i]t}^{\left(pre\right)}$ denotes the pre-spike membrane potential for neuron *i* at time bin *t*, ${\hat{x}}_{t}^{\left(pre\right)}=\left(1-\Delta \frac{1}{\tau }\right)Wr_{t-1}$ denotes the network output for the current time bin *before spiking*, and *v*_[*i*]*t*−1_ = **w**_*i*_^⊤^(**z**_*t*−1_ − *W* **r**_*t*−1_) denotes the (post-spike) membrane potential from the previous time step.

Second, spikes for the current time bin are computed by determining whether pre-spike membrane potential exceeds threshold ($v_{[i]t}^{\left(pre\right)}>T_{i}$). When this occurs, the neuron records a spike: *s*_[*i*]*t*_ = 1.

Lastly, the filtered spike trains *r*_[*i*]*t*_ are augmented and membrane potential is reset:

$$\begin{array}{cccc} r_{[i]t} & =\left(1-\Delta \frac{1}{\tau }\right)r_{[i]t-1}+s_{[i]t}\; & & \left(\text{filtered}\;\text{spike}\;\text{train}\;\text{update}\right) \end{array}$$ (11)

$$\begin{array}{cccc} v_{[i]t} & =w_{i}{}^{⊤}\left(z_{t}-{\hat{x}}_{t}\right)=v_{[i]t}^{\left(pre\right)}-w_{i}{}^{⊤}Ws_{t},\; & & \left(\text{post}-\text{spike}\;\text{membrane}\;\text{potential}\right) \end{array}$$ (12)

which ensures that post-spike membrane potential equals the difference between the projected proxy variable and network output.

#### Vector update rules

For convenience, we can rewrite the BSN update equations in vector form. The pre-spike membrane potential is given by:

$$\begin{array}{cc} v_{t}^{\left(pre\right)} & =W{}^{⊤}\left(z_{t}-{\hat{x}}_{t}^{\left(pre\right)}\right)\\ & =v_{t-1}+\Delta W{}^{⊤}\left(A+\frac{1}{\tau }I\right)Wr_{t-1}+\Delta W{}^{⊤}c_{t}\\ & =v_{t-1}+\Delta (\Omega r_{t-1}+W{}^{⊤}c_{t})\;\left(\text{vector}\;\text{pre}-\text{spike}\;\text{membrane}\;\text{potential}\right) \end{array}$$ (13)

where $\Omega =W{}^{⊤}\left(A+\frac{1}{\tau }\right)W$ are the coupling weights from **r**_*t*−1_ to the pre-spike membrane potential, which implement computation of the divergence between the target **z**_*t*_ and passive decay of **r**_*t*_ in the absence of spiking.

Once the spike vector **s**_*t*_ has been computed, the filtered spike trains and network output for the current time bin are given by:

$$\begin{array}{cccc} \;\;r_{t} & =\left(1-\Delta \frac{1}{\tau }\right)r_{t-1}+s_{t} & \; & \left(\text{vector}\;\text{of}\;\text{filtered}\;\text{spike}\;\text{trains}\right) \end{array}$$ (14)

$$\begin{array}{cccc} \;\;{\hat{x}}_{t} & =Wr_{t}, & & \left(\text{vector}\;\text{network}\;\text{output}\right) \end{array}$$ (15)

and the vector of post-spike membrane potentials is given by

$$\begin{array}{cccc} v_{t} & =W{}^{⊤}\left(z_{t}-{\hat{x}}_{t}\right)\\ & =v_{t}^{\left(pre\right)}-W{}^{⊤}Ws_{t}\\ & =v_{t-1}+\Delta (\Omega r_{t-1}+W{}^{⊤}c_{t})-W{}^{⊤}Ws_{t}, & & \left(\text{vector}\;\text{post}-\text{spike}\;\text{membrane}\;\text{potential}\right) \end{array}$$ (16)

which reflects reset of the pre-spike membrane potential after spiking, but can equivalently be seen to be the projected difference between the proxy variable **z**_*t*_ and the current network output ${\hat{x}}_{t}$ in the current time bin.

It is worth noting that this model requires the instantaneous propagation of spikes between neurons. After a spike, the membrane potential reset (Eq 16) updates **v**_*t*_ for all neurons based on the spikes in the current time bin via the fast weights, −*W*^⊤^*W*. Although Boerlin *et al* refer to the weights −*W*^⊤^*W* as “fast synapses” and the Ω as “slow synapses”, note that the Ω **r**_*t*−1_ term also involves near-instantaneous propagation of information, since the exponentially-filtered spike trains **r** jump by 1 after every spike.

The full BSN model first described in [31] contained additional penalties on **r**_*t*_ in the objective function, which had the effect of reducing spiking by trading off minimization of error (Eq 4) against a cost of inserting spikes. Although we have left these terms out our derivation here for simplicity, including them has the limited effect of changing the spike threshold and post-spike reset and does not change the nature of our findings. The simulations of the original BSN shown in the following sections use the full version as described in the Methods section. Simulation parameters for each figure are also included in the Methods section.

### Limitations of the BSN model

A key limitation of the BSN model is that it requires unrealistically fast communication between neurons. In the standard integrate-and-fire model, a spike resets only the membrane potential of the neuron that emitted it. In the BSN model, by contrast, the membrane potentials of *all* neurons reset following a spike from *any* neuron via the −*W*^⊤^*W***s**_*t*_ term in (Eq 16). The instantaneous reset following a spike is necessary to ensure that each neuron’s membrane potential maintains an accurate representation of the read-out after each spike. From a normative standpoint, the hard LIF threshold entails that maintaining an accurate local copy of the error is critical for the network to encode the target.

In fact, the problem is slightly worse than this: standard implementations of the BSN model include a constraint that only one neuron is allowed to spike in a single time bin as a way of imposing instantaneous (i.e., sub-time bin) communication. Without this constraint, neurons with similar output weights tend to spike in the same time bin, when a spike from any one of them would have sufficed to compensate for error in network output. This causes the network output to dramatically overshoot the target. In the subsequent time bin, neurons with opposite-sign weights fire to compensate for this error, and overshoot the target by a large amount in the opposite direction. This sets up a pathological pattern of oscillatory firing known as “ping-ponging”, in which two populations spike maximally in alternating time bins of the discrete simulation [31, 36].

**Fig 2:**
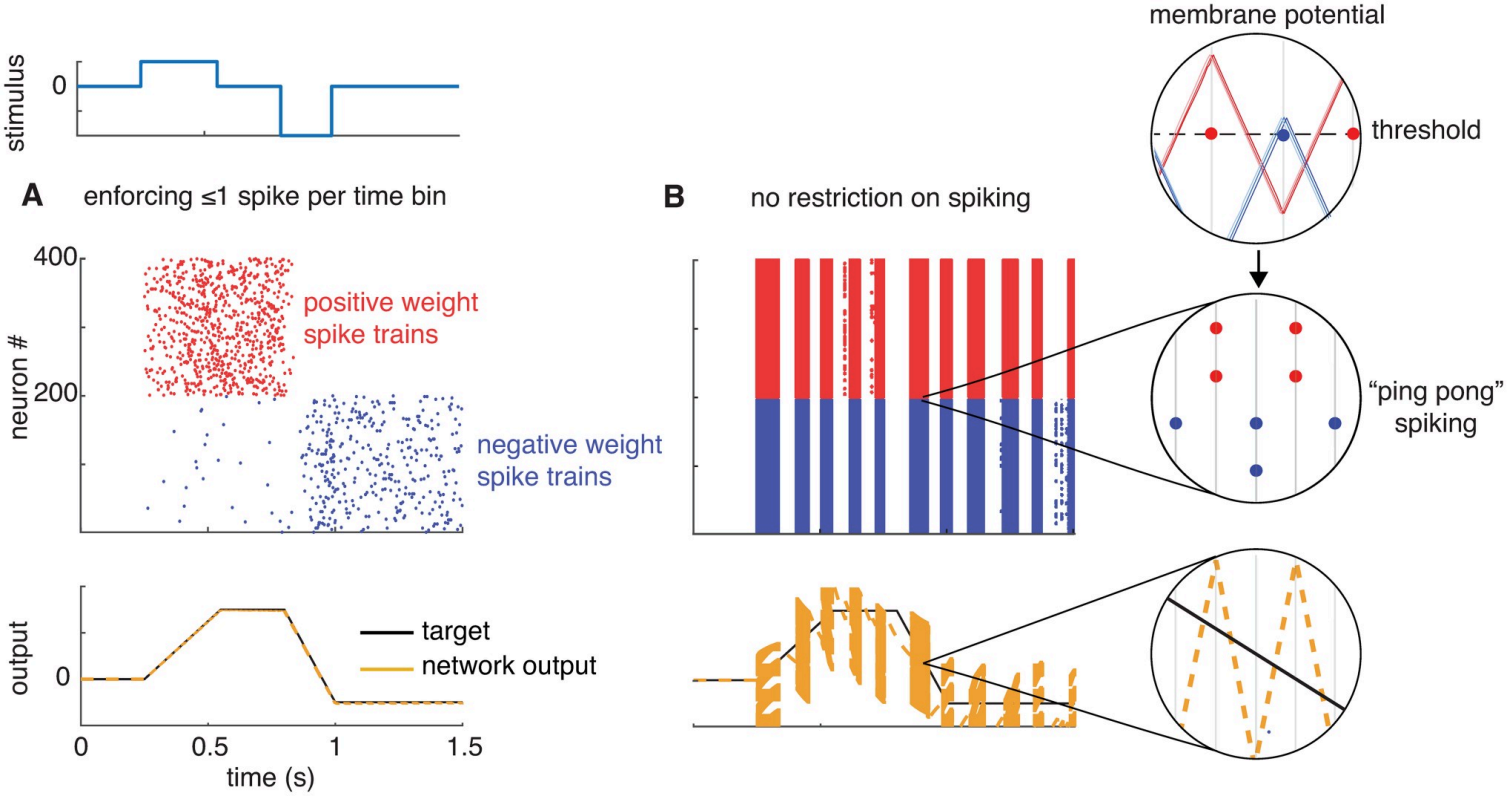
Balanced spiking network implementing an exact integrator. The network consists of 400 neurons, divided into two populations with output weights of + 0.1 (red) and −0.1 (blue). **(A)** Simulation results under the condition that only one neuron is allowed to fire per discrete time bin. **(B)** Simulation results when all neurons whose membrane potential is above threshold in a single time bin are allowed to fire, leading to “ping-pong” behavior. Insets show that the read-out (yellow) is alternating between large over- and under-estimates of the target (in black). Insets show, in order from top to bottom: the voltage traces of neurons in both positively and negatively weighted populations for a small time window, the resulting spikes in each time bin, and the resulting read-out (yellow) and target (black). Since the weights and inputs are identical across populations, so are the voltage traces. Ping-ponging results, as all neurons within a population cross the threshold in the same time bin, spike, and cause the read-out to oscillate between over- and under-estimates of the target.

Fig 2 illustrates how ping-pong behavior can arise if multiple spikes are allowed in a single time bin. We set the BSN model to implement a 1-dimensional exact integrator, $\dot{x}\left(t\right)=c\left(t\right)$, using the same parameters as the example from figure 1C of [31]. In brief, the network contained 400 neurons, divided into two equal sized populations with output weights of +0.1 and -0.1, respectively, which we refer to as positive-output and negative-output neurons (see Methods for complete details). When the rule forbidding multiple spikes per time bin is imposed, the network accurately tracks the target output variable (Fig 2A). However, removing this constraint—allowing all neurons with membrane potential above threshold to fire—results in ping-pong behavior and large errors in tracking the target (Fig 2B).

Note that ping-ponging could be eliminated by computing threshold-crossing times with extremely high temporal precision, either using very small time bins or spike-time interpolation methods [37, 38]. Such precision may allow us to identify the first neuron to spike and to near-instantaneously reset the membrane potential of other neurons, preventing overshoot of the target. However, this near-instantaneous reset of the membrane potential in other neurons is at odds with the timescale of synaptic communication, and represents a shortcoming of the original BSN framework that our work seeks to overcome. (See Discussion for an overview of other approaches to the problem of instantaneous synapses, e.g. [36]). In the following sections, we imposed the constraint that only one neuron could spike per time bin for all simulations of the original BSN model, consistent with [31].

### BSN with conditionally Poisson neurons

To overcome the problems of instantaneous transmission and network instability, we propose two novel formulations of balanced spiking networks with conditionally Poisson neurons: (1) a local framework, where each neuron spikes independently based on its local estimate of network error; and (2) a population-level framework, which sets firing rates to reduce expected error for the entire population.

The key idea in both frameworks is to replace the deterministic integrate-and-fire spiking rule with probabilistic spiking. Under this modified spiking rule, spiking is governed by an instantaneous probability of spiking λ_*t*_, also known as the *conditional intensity*, such that spiking is independent with probability Δλ_*t*_ in any small time window of width Δ. This results in an auto-regressive Poisson generalized linear model (GLM), also known as a Cox process [33–35]. This model has a quasi-realistic biophysical interpretation [32, 39, 40], and recent work has shown that it can capture a wide range of dynamical behaviors found in real neurons [35].

#### Local framework

**Fig 3:**
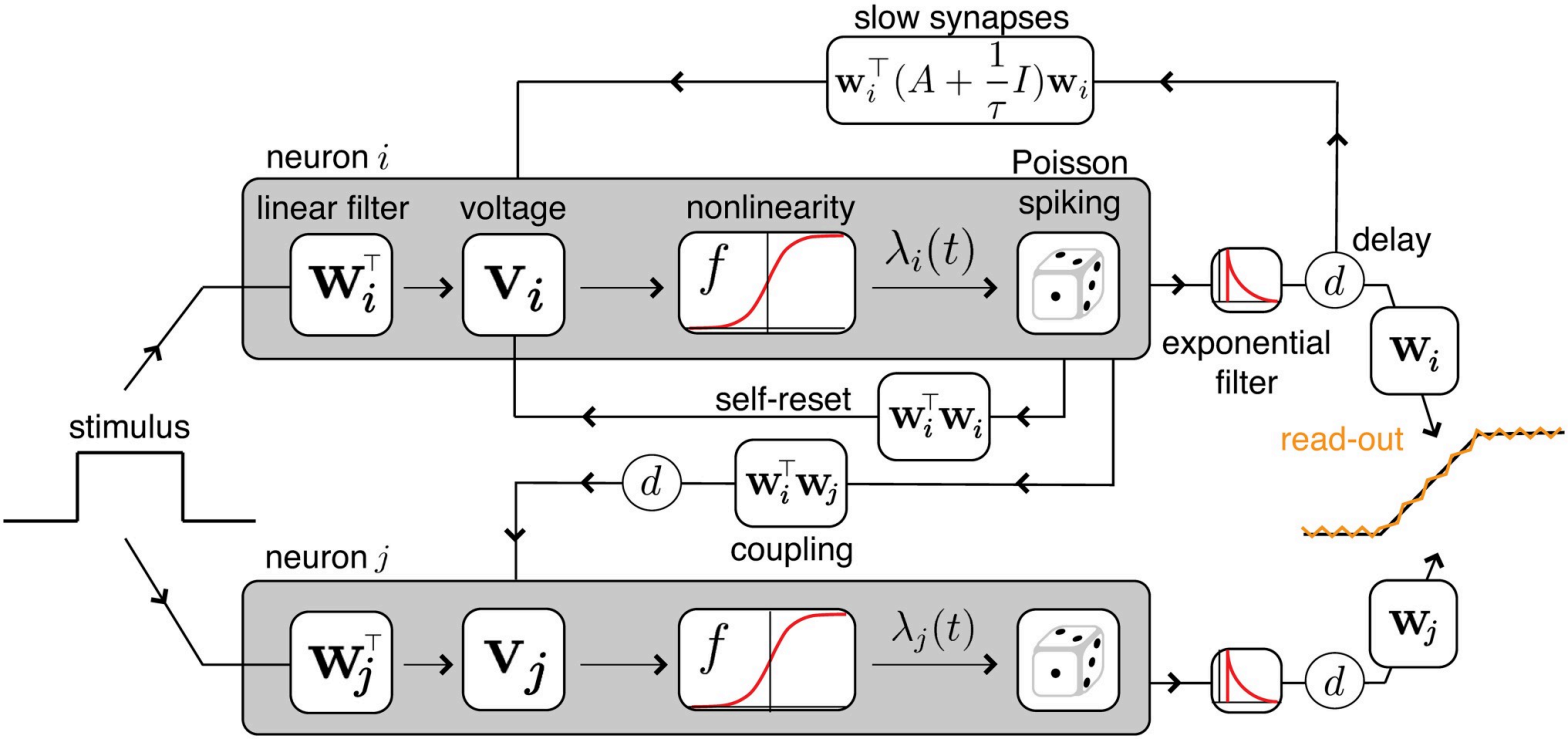
Schematic of two neurons in a BSN with conditionally Poisson neurons. The stimulus influences each neuron’s membrane potential **v**_*i*_ via a set of input weights *W*^⊤^. The neurons reset themselves via instantaneous, fast synapses. Fast connections to other neurons propagate the effects of spikes with a synaptic time delay *d*. The desired linear dynamics are implemented via slow weights (through spike trains filtered by an exponential) also with a time delay *d*. Within each neuron, spiking is probabilistic with an instantaneous probability of firing λ_*i*_(*t*) = *f*(*v*_*i*_(*t*)), where *f*(⋅) is a nonlinear function of voltage. Self-connections are only shown for neuron i.

A simple way to introduce probabilistic spiking to the BSN framework is to replace the hard spike threshold of the integrate-and-fire model with a soft threshold, so that spike probability grows as a nonlinear function of membrane potential, an approach also known as the “escape-rate approximation” [32]. Specifically, we define each neuron’s conditional intensity function to be a sigmoidal function of membrane potential:

$$\begin{array}{c} \lambda_{t}=f\left(v_{t}\right)=\frac{F_{max}-F_{min}}{1+e^{-\alpha (v_{t}-T)}}+F_{min},\;\left(\text{nonlinearity}\right) \end{array}$$ (17)

where *v*_*t*_ is the membrane potential at time *t*, *T* is the spike threshold, *α* is a slope parameter governing the sharpness of the threshold, *F*_*max*_ is the maximal firing rate and *F*_*min*_ is a baseline firing rate, meant to simulate random firing activity in the absence of a stimulus. The probability of spiking in a small time interval is proportional to λ_*t*_, which models the spike response as an inhomogenous Poisson process. We refer to this as the local Poisson framework because, like the BSN, spikes are generated by internal dynamics that evolve according to local copies of the representation error. See Fig 3 for a schematic.

**Fig 4:**
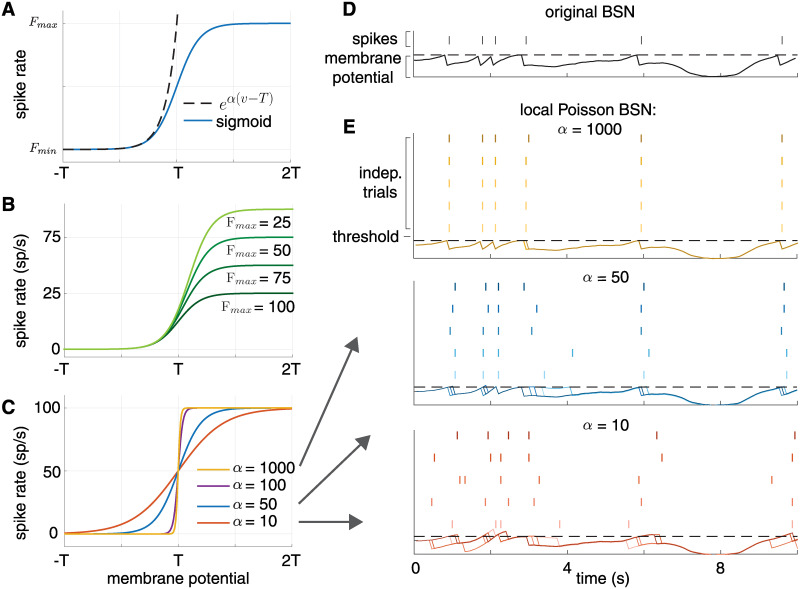
**(A)** The conditional intensity for the exponential non-linearity (dashed lines) and the sigmoid non-linearity (solid lines). The conditional intensity of the sigmoidal non-linearity closely follows that of the exponential non-linearity for sub-threshold voltages, but levels off after threshold, keeping firing rates stable. **(B)** Family of nonlinearities with varying *F*_*max*_. Increasing *F*_*max*_ raises the firing rate at which the nonlinearity saturates. **(C)** Family of nonlinearities with varying *α*. Increasing *α* increases the steepness of the nonlinearity, which approaches a hard-threshold function as *α* → ∞ (like the BSN). **(D)** Simulation of the original BSN implementing an exact integrator, showing membrane potential and spikes of a single example neuron. **(E)** Spikes and membrane potential of the same neuron in a local Poisson BSN implementation of the same system. High *α* simulations (yellow) replicate the behavior of the BSN integrator. Lowering *α* to 50 (blue) or 10 (red) results in a spread of spikes centered around the deterministic BSN spikes.

Although it is common to use exponential nonlinearities for Poisson GLMs, here we have used a scaled sigmoid function to control both the suddenness of firing onset and the maximum achievable firing rate. The parameter *α* controls the precision of firing onset, while *F*_*max*_ and *F*_*min*_ control the range of firing rates within a small time window. The resulting function (Eq 17) resembles an exponential function at low firing rates but saturates at a maximum of *F*_*max*_ (see Fig 4). If we let both *α*, *F*_*max*_ → ∞, we recover the (hard-threshold) integrate-and-fire rule of the original BSN, in which a spike occurs probability 1 when *v*_*t*_ > *T*. However, for finite *α* and *F*_*max*_, the onset of spiking is more gradual.

As a practical matter, we do not wish to allow a single neuron to fire multiple spikes in a single time bin, because the first spike would preclude additional spikes in the same time bin due to “reset” of the membrane potential. We therefore simulate the model with the spiking rule:

$$\begin{array}{c} P\left(s_{t}=1|\lambda_{t}\right)=1-\exp\left(-\Delta \lambda_{t}\right),\;\left(\text{local}\;\text{Poisson}\;\text{framework}\;\text{firing}\;\text{rule}\right) \end{array}$$ (18)

where exp(−Δλ_*t*_) is the probability of observing no spikes in a time bin of size Δ under the Poisson model. For each time-step, we update **v** as in the original BSN model, pass it through the nonlinear function *f*(⋅) to obtain the vector of Poisson firing rates, λ_*t*_, and draw spikes as independent Bernoulli random variables with probability as given above.

Fig 4 illustrates how *α* affects spiking precision by comparing spike times of a single neuron integrating a noisy stimulus implementing the original BSN framework (Fig 4D) to the local framework with different values of *α* (Fig 4E). As we expect, for high values of *α* we recover the precise spiking behavior of the BSN model. As *α* decreases, spike times spread around the ideal BSN spikes. Note that the precise amount of spikes fired in the time window (four) is conserved, although the timing is highly variable. On average, though, the local framework has spike times centered around those of the original BSN.

#### Robustness to parameters of the nonlinearity

**Fig 5:**
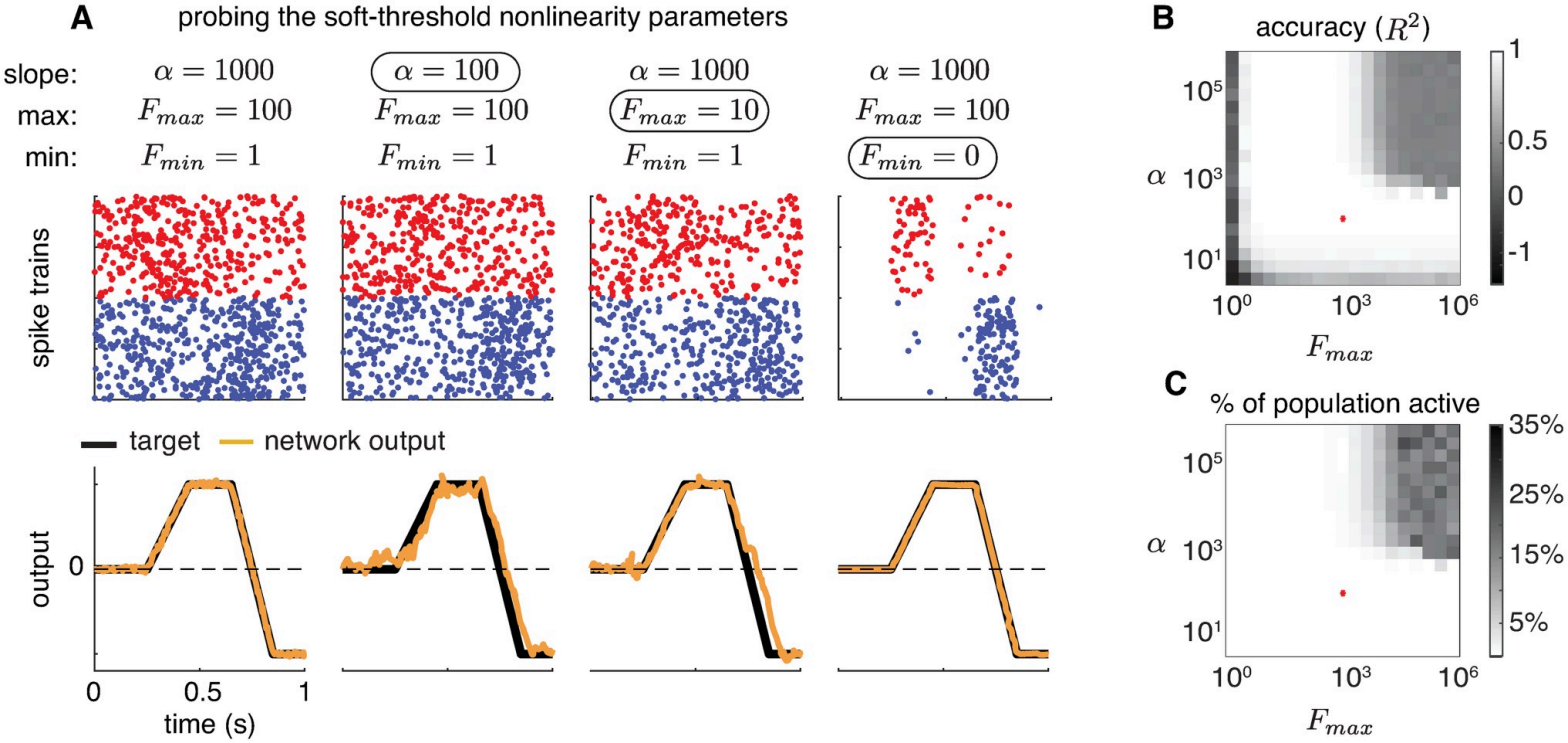
**(A)** Simulations of local Poisson model showing the effects of varying the parameters of the soft-threshold nonlinearity on performance. Relevant parameters are the slope *α*, maximal firing rate *F*_*max*_, and baseline firing rate *F*_*min*_. Network dynamics implemented an exact 1D integrator and the stimulus was the same as Fig 2. Red and blue dots indicate spikes from neurons with positive and negative output weights, respectively. **(B)** Network performance as quantified by *R*^2^ across a range of parameter settings with baseline fixed at *F*_*min*_ = 0 (log-scale). Red asterisk indicates the values for the rightmost column of A (*α* = 1000, *F*_*max*_ = 100). Accuracy remains high across a broad range of parameter values, falling substantially below 1 when slope and maximum firing rate are both large or very low. **(C)** Percent of the neural population active as a function of *α* and *F*_*max*_, with *F*_*m*_ *in* = 0. The network shows ping-ponging behavior in upper right corner, where the model approaches a deterministic, hard-threshold firing rule.

We studied the effects of varying *α*, *F*_*max*_, and *F*_*min*_ on the performance of the homogeneous integrator network (Fig 5) and found that there exists a wide range of values for which the network error is low and the spiking activity is efficient (Fig 5B and 5C). The rightmost panel on Fig 5A shows raster plots and corresponding read-outs for ‘optimal’ parameter settings (defined as being in the low error and activity range, denoted by the red * in Fig 5B and 5C), and in the subsequent panels we modify each of the parameters in turn to show qualitative changes in spiking activity and read-out accuracy. Decreasing *α* and *F*_*max*_ negatively impacts read-out quality, while removing background spiking returns the network to a high-precision, synchronized regime.

As shown in figure (Fig 5B), network performance noticeably deteriorates for very large values of *α* and *F*_*max*_. This happens because we are forcing the precision to be too high through *α* while allowing neurons to spike too frequently through *F*_*max*_. Since the spiking rule is localized, large proportions of the population are active at the same time (Fig 5C), much what happens when the original BSN starts to ping-pong. However, the values at which this happens (e.g., *F*_*max*_ > 10^3^ spikes/second) are well above what we would expect to see in a biological system. Likewise, we observe a low *R*^2^ value when *α* and *F*_*m*_*ax* are very low. In this case, the probability of spiking is simply too small for spikes to be fired in a given time bin, despite large coding errors. Although these results are using relatively simple target dynamics, we still observe a wide ranges of stable *F*_*max*_ and *α* settings for more complex or multi-dimensional simulations.

The computational advantage of our probabilistic spiking rule is that it introduces uncertainty into spike timing, therefore preventing all neurons from firing at once. The parameters controlling this asynchrony, *F*_*max*_, *F*_*min*_ and *α*, have direct physical interpretations (maximal and minimal/background firing rate and error tolerance, respectively) which can be mapped on to characteristics of real neural circuits.

**Fig 6:**
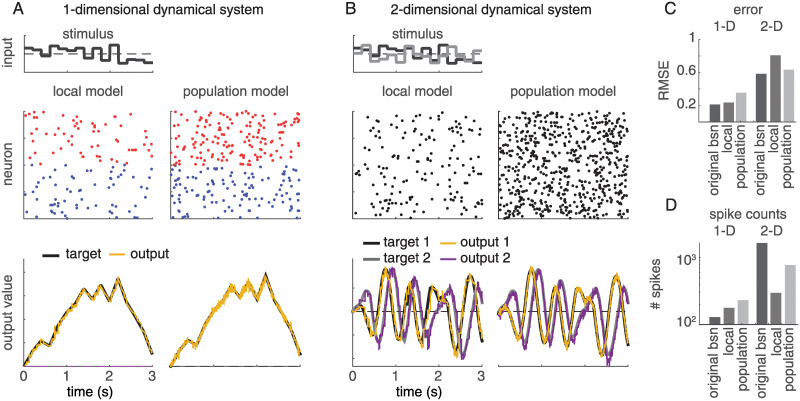
Simulations of the local and population frameworks implementing a 1D and 2D dynamical system. (**A**) The target was a 1-dimensional integrator: $\dot{x}\left(t\right)=c\left(t\right)$. Left side shows spikes and outputs from local Poisson model, while right side shows spikes and outputs for population Poisson model. As in previous figures, red dots indicate spikes from neurons with positive output weights, blue dots indicate spikes from neurons with negative weights. (**B**) The target was a 2-dimensional oscillator ${\dot{x}}_{1}\left(t\right)=-x_{1}\left(t\right)-10x_{2}\left(t\right)+c\left(t\right);x_{2}\left(t\right)=10x_{1}\left(t\right)-x_{2}\left(t\right)+c\left(t\right)$. For the population model, the time window for computing expected spike count was *κ* = 5ms (50 time bins). Weights were randomized to be positive or negative in either dimension, such that neurons are no longer divided into strictly positive- or negative-weight groups. **(C)** Accuracy (measured by root-mean-squared error) of the two models for 1D and 2D systems. (**D**) Number of spikes emitted by each model during simulations (log scale).

**Fig 7:**
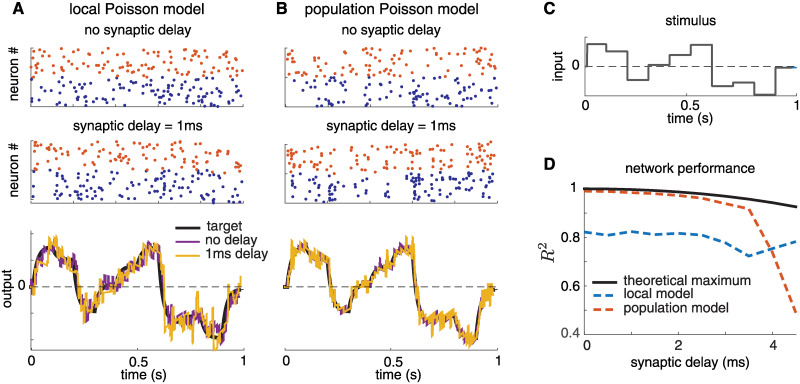
Illustration of local and population conditionally Poisson BSN frameworks with synaptic delays. **(A)** Spike trains simulated from the local Poisson framework implementing a 1D exact integrator, both without (top) and with a 1-ms synaptic delay (middle). The network output accurately tracked the target variable for both models (bottom). As before, red/blue spike trains indicate neurons with positive/output weights. **(B)** Analogous plots for population Poisson framework. **(C)** Stimulus used for simulations shown in A and B. **(D)** Coefficient of determination (*R*^2^) computed using 50 simulations of each framework. Black trace indicates the maximum possible *R*^2^ value that could be obtained given the exponential Euler integration rule (see Methods).

In theory, a BSN network with voltages corrupted by additive Gaussian noise could behave similarly to the local framework. However, we found that this model still tended to exhibit ping-ponging unless noise amplitude approached the amplitude of signal-induced fluctuations in membrane potential, which adversely affected coding accuracy. By contrast, the local framework is stable even when the resets of other neurons is delayed to the next time bin (see Figs 5 and 6) or by several milliseconds (Fig 7). Intuitively, the reason for this is that, in addition to the slope parameter alpha, which controls the steepness of the soft-threshold-crossing process (in a way, similar to additive voltage noise in the original BSN), we have a limit on the maximal firing rate *F*_*max*_, which ensures that there will not be too many spikes across the population even when many neurons are driven simultaneously across their (noisy) threshold. That is, it would take several time bins for them all to fire given the value of *F*_*max*_, which is enough time for synaptic inhibition to arrive and prevent firing. Moreover, in Fig 5B we show that there is a broad range of parameters over which the local population is able to achieve stable, accurate coding. Strictly speaking, by introducing extra degrees of freedom, we are losing the strict normative angle of the original BSN framework. However, what we gain in the process—a considerably expanded space of stable network configurations—makes it possible to introduce realistic communication delays between neurons.

### Population framework

We now describe a second framework for implementing BSNs with conditionally Poisson neurons. In this approach, we take a population-level instead of a neuron-level view of the optimization problem to be solved. Instead of assigning each neuron to carry an independent representation of the error between the target and actual network output, we assign each neuron an analog probability of firing such that expected number of spikes across the network compensates appropriately for the total error. We refer to this as the population framework.

The derivation of this framework starts from the same foundation of the original BSN, namely, an error function describing the discrepancy between target and actual network output. However, instead of specifying that each neuron should spike whenever doing so will reduce error (Eq 5), we compute a vector of spike rates λ, such that the expected spike response across the population over some time window of length *κ* will minimize error. This leads to the following network objective function:

$$\begin{array}{c} E_{t}=\left|\right|x_{t}-\left({\hat{x}}_{t}+\kappa W\lambda \right){\left|\right|}_{2}^{2},\;\left(‘{\text{population}}^{’}\;\text{error}\;\text{function}\right) \end{array}$$ (19)

where **x**_*t*_ is the target output at time *t*, ${\hat{x}}_{t}$ is the actual network output at time *t*, *W* are the decoding weights, and **λ** is the vector of firing rates (conditional intensities) of a network of Poisson spiking neurons. In this expression, *κW* **λ** is expected contribution to network output over a time window of size *κ*. For implementation in discrete time, *κ* should be an integer multiple of the bin size Δ.

To minimize the above error, we set the instantaneous spike rate vector equal to the least-squares solution:

$$\begin{array}{c} \lambda_{t}=\frac{1}{\kappa }\tilde{W}\left(x_{t}-{\hat{x}}_{t}\right) \end{array}$$ (20)

where $\tilde{W}=W{}^{⊤}{\left(WW{}^{⊤}\right)}^{-1}$ is the Moore-Penrose pseudo-inverse of the decoding weight matrix *W*. Poisson neurons firing independently with conditional intensity **λ**_*t*_ will therefore minimize the expected error between target and actual network output. Note that if *W* has orthogonal unit-vector rows, such that *WW*^⊤^ = *I*, then $\tilde{W}=W{}^{⊤}$ and we obtain the same encoding weights as the original BSN framework. For the implementation of the population framework, we make the same substitution of the proxy variable **z** for **x**.

In the local framework, increasing the population size means more neurons are competing to reduce the read-out error in a single time window. This increased activity can lead to ping-ponging. The population level view of the problem scales the probability of spiking, **λ**_[*i*]_, by *WW*^⊤^, which increases with population size. This re-scaling effectively spreads the ‘responsibility’ of correcting an error across the entire population by incorporating information about the total network size in an individual neuron’s activity.

However, the solution in (Eq 19) is not valid generally because the right-hand-side can take on negative values, whereas the conditional intensity for a Poisson process must be positive. To overcome this, we create two mirrored copies of the population. Positive firing rates are assigned to one copy with weight vector *W*, and negative firing rates are assigned to the other copy with weight vector −*W*. We distinguish between these neurons and ‘anti-neurons’ by the sign of their membrane potential as determined by the least squared solution. Similarly to the local and BSN models, spikes from either population will have opposite effects on the output variable. However, in this case these designations are not fixed labels and do not apply to the actual sign of **w**_*i*_. For example, spikes from the *i*’th neuron will contribute **w**_*i*_ to the network output, while a spike from its ‘anti-neuron’ counterpart will have a contribution of −**w**_*i*_ to network output, but the entries of **w**_*i*_ themselves may be positive or negative.

Formally, we define the population framework in terms of the update equations:

$$\begin{array}{ccc} v_{t} & =\tilde{W}\left(z_{t}-{\hat{x}}_{t}\right) & \\ & =v_{t-1}+\Delta (\tilde{\Omega }r_{t-1}+\tilde{W}c_{t}) & \left(\text{pre}-\text{spike}\;\text{membrane}\;\text{potential}\right) \end{array}$$ (21)

$$\begin{array}{cccc} \lambda_{t}^{\left(+\right)} & =\frac{1}{\kappa }\text{max}\left(v_{t},0\right) & & \left(\text{neuron}\;\text{spike}\;\text{rate}\right) \end{array}$$ (22)

$$\begin{array}{cccc} \lambda_{t}^{\left(-\right)} & =\frac{1}{\kappa }\text{max}\left(-v_{t},0\right) & & \left(\text{anti}-\text{neuron}\;\text{spike}\;\text{rate}\right) \end{array}$$ (23)

$$\begin{array}{cccc} s_{t}^{\left(+\right)} & ∼\text{Poiss}(\Delta \lambda_{t}^{\left(+\right)}) & & \left(\text{neuron}\;\text{spikes}\right) \end{array}$$ (24)

$$\begin{array}{cccc} s_{t}^{\left(-\right)} & ∼\text{Poiss}(\Delta \lambda_{t}^{\left(-\right)}) & & \left(\text{anti}-\text{neuron}\;\text{spikes}\right) \end{array}$$ (25)

$$\begin{array}{cccc} r_{t} & =\left(1-\Delta \frac{1}{\tau }\right)r_{t-1}+s_{t}^{\left(+\right)}-s_{t}^{\left(-\right)}\; & & \left(\text{population}\;\text{filtered}\;\text{spike}\;\text{rates}\right) \end{array}$$ (26)

where $\tilde{\Omega }=\tilde{W}\left(A+\frac{1}{\tau }\right)W$ are the coupling weights from **r**_*t*−1_ to the pre-spike membrane potentials. This differs from the standard BSN framework in that spikes, rather than being driven by deterministic threshold crossing, arise from a Poisson process with conditional intensity $\lambda_{t}^{\left(+\right)}$ or $\lambda_{t}^{\left(-\right)}$. If **v**_[*i*]*t*_ is negative, then $\lambda_{[i]t}^{\left(+\right)}$ is set to zero, and the corresponding anti-neuron’s firing rate is positive. The voltage updates and spiking resets are identical to the local and BSN models.

Fig 6 shows a comparison of the activity of the local and population-level frameworks, as well as the accuracy and spike counts between all three models. Fig 6A and 6B show that the local and population models perform similarly, although the population framework has a higher spike rate. Between the three models, the original BSN model has a lower decoding error than the local and population Poisson models for both the one-dimensional and two-dimensional dynamical systems (Fig 6C), although at the expense of a greatly increased number of spikes (Fig 6D). This is due to the deterministic spike rule, which enforces that any spike fired must perfectly compensate for the error in every given time bin (or as well as is allowed by the size of the decoding weights, *W*). Spiking in the local and population frameworks is *driven* by this requirement, but is stochastic.

However, in terms of accuracy, all three models had similar *R*^2^ values: 0.9961 (BSN), 0.9957 (local), and 0.9928 (population) for the 1-D and 0.9686 (BSN), 0.9395 (local) and 0.9565 (population) for the 2-D dynamical system. Thus, the probabilistic frameworks, on average, compensate for the error as well as the original BSN. But in any given time bin, there is a certain probability of firing too early or too late to optimally compensate for the error, which results in a higher RMSE. Note that the performance of the local and population models depends on the parameters of the firing rate nonlinearity and on *κ*, respectively, and modifying those can move the models into lower-error (and higher spike count) regimes.

### Incorporating synaptic time delays

The original BSN model relies on near-instantaneous synaptic communication between neurons since all neurons in the population must reset immediately after a spike in any neuron. A more realistic model would require that synaptic inputs arrive only after a brief synaptic delay; only the reset of a neuron’s own membrane potential following a spike could be considered instantaneous.

To test the robustness of the two Poisson BSN frameworks introduced above, we altered synaptic currents to incorporate a synaptic delay in neural outputs. In the revised model, filtered spike trains and “fast” membrane potential resets are received by other neurons only after a synaptic delay *d*. Thus, if neuron fires at time *t*, it resets its own membrane potential in the next time bin, but we will update spike trains and filtered spike trains received by other neurons only at time *t* + *d*.

To compensate for synaptic delays, we altered the network dynamics so that membrane potential—instead of reflecting the *current* error, as in the original BSN— reflects the network error extrapolated *d* time steps into the future. The basic logic of this approach is that the network dynamics should look into the future to predict whether firing a spike now will reduce error at the time when the spike actually arrives. The resulting solution is equivalent to minimizing an objective function (Eqs 4 or 19) where **x**_*t*+*d*_ and ${\hat{x}}_{t+d}$ take the place of **x**_*t*_ and ${\hat{x}}_{t}$.:

$$\begin{array}{cc} v_{t} & =W{}^{⊤}\left(z_{t+d}-{\hat{x}}_{t+d}\right),\;\left(\text{membrane}\;\text{potential}\;\text{with}\;\text{synaptic}\;\text{delay}\right) \end{array}$$ (27)

Here, $z_{t+d}=\exp\left(Ad\right)z_{t}+\frac{c}{A}\left(e^{Ad}-1\right)$ and ${\hat{x}}_{t+d}=\exp\left(-d/\tau \right){\hat{x}}_{t}=W\exp\left(-d/\tau \right)r_{t}$ represent the extrapolated target and network outputs at time *t* + *d*, respectively. This solution arises by analytically solving the differential equation $\frac{dz}{dt}=Az+c$, which describes the target dynamics, for **z** at time *t* + *d* given an initial condition **z**_*t*_ and *c* = *c*(*t*), and solving the equation $\frac{dr}{dt}=-\left(1/\tau \right)r$, which describes passive network dynamics in the absence of spiking, for **r** at time *t* + *d* given an initial condition **r**_*t*_. For the population-level Poisson framework, the encoding weights $\tilde{W}$ replace *W*^⊤^ in (Eq 27). (See Methods for details).

Fig 7 shows an analysis of the accuracy of the local and population-level Poisson frameworks with synaptic delays. For both models, a 5ms synaptic delay does not have pronounced effects on the spiking activity or the quality of the read-out (Fig 7A and 7B). Fig 7D shows the *R*^2^ values as a function of time delay for both frameworks. We compare it against a theoretical upper bound on the coding accuracy (in black), which is a consequence of the exponential Euler approximation (see Methods for details).

For the local framework, the *R*^2^ value is lower than for the simulation shown in Fig 6 because the parameters were chosen to make the network more robust to synaptic delays. Otherwise, in the high-precision (high *α*) regime with synaptic delays, ping-ponging may result, as shown by the higher error regime in Fig 5B. By contrast, the population framework can maintain high levels of accuracy for a large range of synaptic delays.

These revised dynamics could also be used to increase accuracy of a standard BSN with synaptic delays, for example as defined in [36]. We attempted to incorporate synaptic delays into our implementation of the standard BSN, but it led to ping-ponging, after which the model was no longer able to track the target dynamics. The resulting *R*^2^ values were negative, so they are not included in Fig 7D.

### Network performance

**Fig 8:**
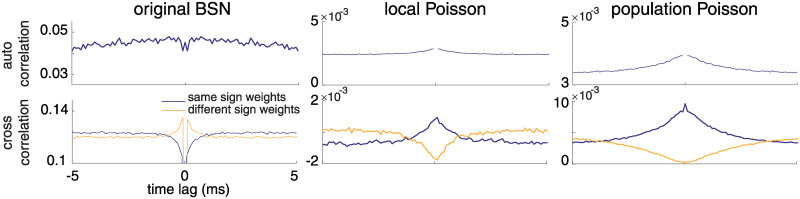
Cross- and auto-correlations for the original BSN, local Poisson and population Poisson BSN models with synaptic delay. The top row shows average auto-correlations across both populations of neurons. The bottom row shows average cross-correlations for pairs of neurons with the same sign output weight (i.e., both positive or both negative, in purple) and for pairs of neurons with opposite-sign output weights (e.g., one positive and one negative neuron, in yellow). The original BSN network exhibits negative correlations between neurons with the same sign, and positive correlations between neurons with opposite sign. The local and Population Poisson models show the opposite pattern, which more closely resembles correlations found in neural populations in (e.g.) visual cortex.

Lastly, we analyzed the performance of our local and population frameworks as compared to the original BSN model. Fig 8 shows the cross-correlations of spike trains generated by a network of forty neurons implementing a one-dimensional integrator, $\dot{x}\left(t\right)=c\left(t\right)$, with a white-noise stimulus *c*(*t*), for the local, population and original BSN models. Local and population Poisson BSN models both enforced a synaptic delay of 1 ms.

To compute cross-correlations, we divided the neurons into positive-output and negative-output groups. We then computed average within-group (positive-positive and negative-negative) and across-group (positive-negative) cross-correlations. These curves show substantial differences between the original BSN model and the two Poisson models. First, cross-correlations of the original BSN model are 0 at lag zero, due to the rule that only one neuron can spike in a single time bin. More importantly, the within-group correlations for the BSN model exhibit a trough at time zero, meaning that neurons with the same output weight are anti-correlated. Conversely, across-group correlations exhibit an increase at small lags, meaning that neurons with opposite sign output weights are more likely to fire together in a small time window. This relationship is at odds with correlations in both retina and visual cortex, where studies have reported that correlations are highest for neurons with similar tuning, and lowest for neurons with dissimilar tuning [34, 41–43]. By contrast, the local and population Poisson models successfully recapitulate this pattern of correlations, with a peak in the cross-correlations between pairs of neurons with the same sign weights, and a trough for pairs of opposite-sign neurons. Cross-correlations from these models also exhibit no trough at zero due to the lack of a rule prohibiting simultaneous spiking. Thus, cross-correlations represent an additional dimension of biological plausibility of the proposed Poisson frameworks.

**Fig 9:**
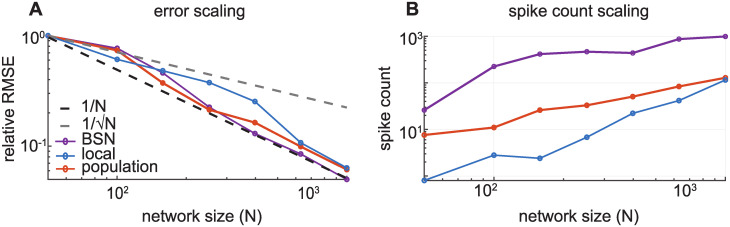
Scaling of error and spike count with population size (weights held fixed). **(A)** Relative root-mean-square error (RMSE) decreases approximately linearly with the network size for all three models. *R*^2^ values for the fit to 1/*N* were .98, .91, and .82 for the BSN, local and population models, respectively. **(B)** Total spike count as a function of network size. The results shown are the average of five simulations of the networks performing exact integration of a signal formed by a sum of two sinusoids.

Fig 9A shows the relationship between root-mean-square error (RMSE) and network size (*N*). The RMSE values shown are *relative* values, normalized in order to best compare the trend in error with respect to *N*. The original BSN model has a decoding error that scales with $\frac{1}{N}$, provided that the weights are likewise scaled by $\frac{1}{N}$, and we find similar scaling for the local and population frameworks, although the BSN has a tighter bound. Poisson rate codes typically scale as $\frac{1}{\sqrt{N}}$ due to the Cramér-Rao bound, which places a lower bound on the variance (and therefore the mean-squared-error) of an unbiased estimator. We might expect the local and population networks to scale similarly.

However, the $\frac{1}{\sqrt{N}}$ scaling applies under the assumption of a constant firing rate. Our local and population models have rates that are a function of voltage dynamics, or the coding error. Although the spiking *mechanism* is Poisson, the conditional intensity is still *driven* by the coding error. So even if the Poisson spikes over- or under-shoot the target in a particular time window, on average the network corrects it in the following bins. In sum, this makes our frameworks’ coding capabilities scale better than $\frac{1}{\sqrt{N}}$.

We also looked at how spike counts varied with network size, shown in Fig 9B. In general, as the weights get smaller, the read-out is more precise, but more spikes are needed to reconstruct the target. The local framework initially has a low spike rate because of the large weights and higher error tolerance (*α* = 1000). However, as the weights get smaller, spiking increases and the local framework exhibits ping-ponging at very small weight sizes. Likewise in the BSN, for small weight sizes the decoding error increases because weights become too small to encode the target given the one-spike rule.

**Fig 10:**
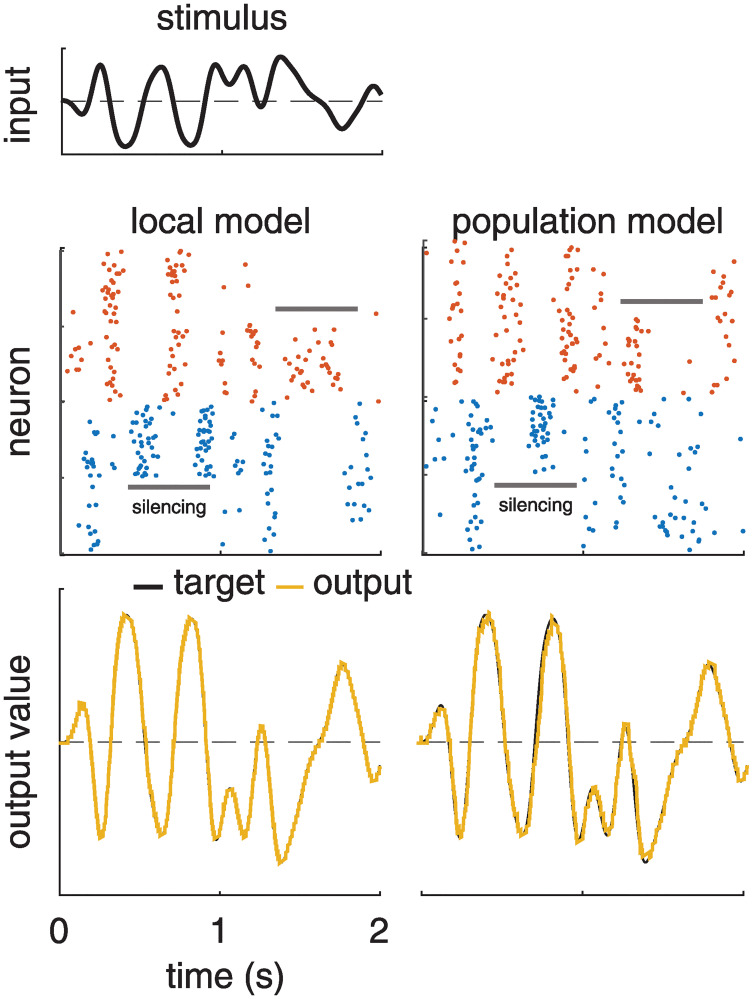
Simulation showing robustness of local and population Poisson models to silencing of a subset of neurons. **Left**: We created a local Poisson BSN model with 400 neurons, with weights set to perform exact integration, and presented it with a slowly varying 1D stimulus (top). From time *t* = .4 to *t* = 1s we artificially silenced 50% of neurons in the negative-weight (blue) population, preventing them from spiking by setting *p*(spike) = 0 (silencing period indicated by the grey bar). From time *t* = 1.2 to *t* = 1.8s we did the same for 50% of neurons in the positive-weight (red) population. **Right**: Likewise for the population framework.

Finally, we looked at how robust both frameworks are to neuron loss. The authors of [31] show in Fig 7G that the original BSN is robust to sudden inactivation of neurons. We ran a similar simulation, shown in Fig 10, silencing 50% of the negatively weighted and positively weighted neurons for.6s at a time by setting the probability of spiking in that time bin to 0. Like in the original BSN, our networks maintain coding accuracy despite large neuron “loss” by increasing the firing rates of remaining neurons.

## Discussion

In this paper, we have highlighted a shortcoming of the balanced spiking network (BSN) paradigm, namely the requirement of near-instantaneous communication between neurons, which arises from the fact that a spike in any neuron causes an instantaneous reset of membrane potential in all other neurons. In practice, the BSN model is often implemented with the additional rule that only one neuron can spike in a single time bin. When synaptic delays are introduced, or multiple spikes are allowed per bin, the model easily enters a ping-ponging regime in which the network output overshoots and undershoots the target output on alternating time bins.

To address this problem, we proposed two extensions to the BSN model that incorporate conditionally Poisson spiking. Our proposed models both preserve the readout structure of the original BSN, in which a linear combination of exponentially filtered spike trains approximates a linear dynamical system of interest. However, they both replace of “hard threshold” integrate-and-fire spiking of the original BSN with a spiking process governed by an instantaneous spike rate or conditional intensity. We note that although conditioning spiking allows for more stable network activity and for synaptic delays, we do so at the expense of the single time bin precision of the original BSN (see, e.g., Fig 6C). The BSN spikes deterministically to correct the coding error in a given time bin, while the local and Poisson frameworks may fire spikes earlier or later than optimal. As such, we relax the guarantee that spikes fired will precisely compensate for the coding error.

In the local Poisson BSN framework, the conditional intensity arises from passing the membrane potential through a sigmoidal nonlinearity. The accelerating phase of this nonlinearity is consistent with nonlinearities observed in neural data [44–47] and closely resembles the exponential nonlinearity commonly used in generalized linear modeling analyses [33, 34], while the saturating phase is consistent with saturation in real neural firing rates.

In the “population” Poisson BSN framework, the conditional intensity is obtained by setting the vector of expected spike counts to the least-squares solution for the total output error. This model differs from the original BSN in that the encoding weights, the linear mapping from output error to membrane potential, uses the pseudo-inverse of the decoding weights, $\tilde{W}$, whereas the original BSN used the transpose *W*^⊤^. This change ensures that the spike rate of each neuron takes account of how many other neurons in the population have similar decoding weights, so that the expected spike count across the entire population in some finite time window compensates optimally for the output error. These modifications make both frameworks robust to both parameter settings and synaptic delays on realistic time scales (1-3ms). They also allow the network to have realistic auto- and cross-correlations, while preserving the decoding accuracy and the robustness of the original BSN.

### Related work

Recent literature has explored a variety of other extensions and applications of the BSN framework, including nonlinear dynamical systems and the learning of synaptic weights [48, 49], synaptic plasticity rules [50], and biological extensions like finite timescale synapses [51] and synaptic delays [52]. The BSN framework has also been adapted to other computational problems such as probabilistic computation [53] and sensory adaptation [54].

Our paper is not the first to address the issue of instability in the BSN. Recent work from [52] examined the use of penalties on spiking to reduce ping-ponging (referred to in that paper as “up states”). We found that this strategy required fine-tuning and succeeded in a relatively narrow parameter regime compared to the solutions we proposed here. Other work by by Chalk et al. [36] has argued that oscillations in the brain activity may arise from BSNs with synaptic delays, suggesting that a substantially damped form of ping-ponging may be a signature of efficient computation in neural circuits. This work used the finite timescale dynamics of [51] to implement the BSN with synaptic delays. Similarly to our work, spiking activity in the Chalk model is asynchronous and sparse, and neurons are not prevented from firing synchronously. However, the Chalk model avoids ping-ponging behavior by injecting a carefully tuned amount of noise into membrane potential and using slow membrane time constants (100ms). The added membrane noise degrades the network representation of coding error, forcing a trade-off between stability and accuracy of representation. By contrast, our models do not require tuning membrane potential noise to achieve stability; although firing is stochastic, the membrane potential maintains an accurate representation of the error over a wide range of input and network sizes. Our network also uses membrane time constants with more biophysically plausible ranges (10-20ms).

The topic of balanced networks has also received considerable attention outside the specific BSN framework introduced by [31]. Balanced networks have been proposed as a substrate for working memory [55, 56], probabilistic inference [57, 58], and the control of complex movements [59]. Excitatory-inhibitory balance is also a key topic in the mathematical theory of neural circuit dynamics, where it has been proposed as an explanation for the correlations found in large-scale population activity [60–62]. Finally, a rich literature has focused on the training of spiking neural networks in more general supervised and reinforcement learning settings, where the objective involves task performance or can only be evaluated at the end of a trial [63–67].

Our population Poisson model is similar to work by Eliasmith and Anderson [68] in that the network objective is to minimize the expected squared error between a target function and a network estimate using a spiking neural network. However, their model does so by optimizing for the decoding weights instead of network activity and uses a spiking mechanism that is driven linearly by the magnitude of the input, instead of the error-correcting computational principle used in the BSN. Later work by Eliasmith [69] extended this framework to allow for embedding of the desired dynamics (i.e., the A matrix) into the population activity.

Our work also connects to a rich literature on point process models of neural spike trains. The local Poisson framework draws direct inspiration from the work of [32], which sought to approximate a noisy integrate-and-fire model with an inhomogeneous Poisson process via the so-called “escape-rate approximation”, which refers to the instantaneous probability of noisy membrane potential crossing threshold in a small time window. Subsequent work on the spike response model [39, 70–73] and Poisson generalized linear model [33–35, 74–76] further explored the connection between integrate-and-fire and conditionally Poisson spike train models. The latter are sometimes referred to as “soft-threshold” integrate-and-fire model [77], making the local Poisson model a natural extension of the original BSN model.

### Future challenges

Although our proposed frameworks are a step in the direction of biological plausibility, there remain a variety of open challenges. A straightforward way of extending the biological realism of our proposed network is to reformulate it with a separate inhibitory population to comply with Dale’s law, as was done in [36]. Another extension is the development of neurally plausible learning rules and weight patterns. The network we proposed has a static weight matrix with all-to-all connectivity. A more realistic model would allow for sparse connectivity, sign constraints forcing neurons to be purely excitatory or inhibitory, and plausible learning rules that allow weights to change over time as a function of reward signals. There have been successes learning the fast, slow and feed-forward weights through non-local, supervised, control theoretic approaches [48, 49] or, much more recently, through local, Hebbian plasticity rules [78]. Our probabilistic formulation of the Poisson BSN frameworks makes implementing local, Hebbian plasticity rules more tractable, as it opens up the possibility of applying unsupervised learning techniques from traditional machine learning methodology.

We note that our implementation of synaptic delays merely shifts the arrival time of spikes but still involves an instantaneous jump in the filtered spike train dynamics. In future work, we hope to implement time delays with a finite rise time, as was done in [51]. We suspect that since our network is stable with instantaneous jumps in **r**(*t*), it will also be stable with smoothly increasing spiking dynamics. Finally, at the level of implementation, we recognize that although our model does not rely on additional spiking conditions or spike-time interpolation, using Poisson firing dynamics instead of the integrate-and-fire approximation represents a slight step away from biological plausibility. We also hope to address the biological plausibility of ‘anti-neurons’ in the population framework.

Another main challenge is the incorporation of nonlinear dynamics. Although the original BSN model was designed to implement linear dynamical systems, it is well known that a wide variety of neural computations are nonlinear. Recent work has proposed an extension of the BSN framework to nonlinear dynamics [30, 48]; combining this approach with conditionally Poisson spiking therefore represents a promising avenue for future work.

Finally, the conditionally Poisson extensions we have proposed provide new opportunities for applying the BSN framework to the interpretation and analysis of real neural data sets. Both the original BSN model and ours assume access to the precise spiking patterns of all the neurons in a population, but real neural recordings typically record only a small fraction of the neurons in a population. Previous work has shown that latent BSN dynamics can be recovered from spike trains in the fully observed case [53]. Other work has discussed the recovery of Poisson generalized linear models from partial recordings [79, 80]. This motivates the development of new methods for identifying balanced network dynamics and computations from partially observed data sets, which may offer fundamental insights into spike-based computation in the brain.

## Methods

### Exponential integrator

Exponential integrators are a class of numerical methods for solving first-order differential equations of the form

$$\begin{array}{c} {\dot{y}}_{t}=-Ay_{t}+g\left(y_{t}\right)+c \end{array}$$ (28)

where −*Ay*_*t*_ is a linear term with a dynamics matrix *A*, *c* is a constant, and the nonlinear terms are grouped in *g*(*y*_*t*_). We use this method of integration throughout our simulations because it is much more numerically stable than explicit Euler methods. For equations without nonlinear terms, it above can be solved exactly from time 0 to a later time *t* as

$$\begin{array}{c} y_{t}=y_{0}e^{At}+\frac{c}{A}\left(e^{At}-1\right) \end{array}$$ (29)

For *A* = 0,

$$\begin{array}{c} y_{t}=y_{0}+ct \end{array}$$ (30)

Using exponential integrators allows us to implement a time delay of magnitude *d* by having the network spike on the basis of an extrapolated future error at a time *t* + *d*. Specifically, we have the network voltage track the error between a predicted **x**(*t* + *d*) and $\hat{x}\left(t+d\right)$. Eq 16 then becomes

$$\begin{array}{cc} v_{t} & =W{}^{⊤}\left(z_{t+d}-{\hat{x}}_{t+d}\right)\\ & =W{}^{⊤}\left(e^{Ad}z_{t}+\frac{c}{A}\left(e^{Ad}-1\right)-e^{-d/\tau }Wr_{t}\right) \end{array}$$ (31)

where we have assumed that the input to the network, *c*(*t*), stays at constant value, *c*, from *t* to *t* + *d* and that the spike rate evolves passively in the absence of spiking. In the case that *A* = 0,

$$\begin{array}{cc} v_{t} & =W{}^{⊤}\left(z_{t}+cd-e^{-d/\tau }Wr_{t}\right) \end{array}$$ (32)

In the case of the population framework, the *W*^⊤^ above is replaced by ${\tilde{W}}^{{}^{⊤}}$.

#### Theoretical minimum error

In Fig 7D we compared the *R*^2^ values for the local and population models as a function of delay to a theoretical upper bound. This bound comes from the expected mismatch between the predicted $x_{t+d}=e^{Ad}y_{t}+\frac{c}{A}\left(e^{Ad}-1\right)$ and the actual value of **x**_*t*+*d*_. To determine this bound, we integrated the target dynamics for the length of the simulation with exponential Euler integration for a range of delays (*d*). We then compared it to the target dynamics integrated without a time delay to get an *R*^2^ for each delay length *d*.

### Simulation parameters

For our simulations, all parameters are non-dimensionalized. Time is measured in seconds and *dt* = 0.1ms. All other simulation parameters are shown in Table 1, below.

**Table 1:** Simulation parameters.

Figure	N	*W*	*α*	*F*_*max*_	*F*_*min*_	*κ*	A	*τ*	*d*
Fig 2	400	± 0.1	N/A	N/A	N/A	N/A	0	10	0
Fig 4D, BSN	1	1	N/A	N/A	N/A	N/A	0	20	0
Fig 4D, GLM	1	1	Varies	50	0	N/A	0	20	0
Fig 5	400	±.1 + noise (*μ* = .01)	1e4	1	0	N/A	0	10	0
Fig 6A and 6B	400	±.1 + noise (*μ* = 1)	1000	1	0	200	0	1	0
Fig 6C and 6D	400	±.1 + noise (*μ* = 1)	1000	1	0	200	$\left(\begin{array}{cc} -1 & -10\\ 10 & -1 \end{array}\right)$	1	0
Fig 7A and 7B	200	± 0.02 + noise (*μ* = .01)	800	100	0	30	-50	0.2	0 or 5ms
Fig 8	40	± 0.0025 + noise (*μ* = .01)	2000	5000	5	20	0	0.5	1ms
Fig 9	varies (50-1000)	$\pm \frac{300}{N}$	1000	2	0.01	1	-100	20	0
Fig 10	400	± 0.2	5000	3	0	50	-100	1	0

Fig 2 was generated using the parameter settings described in Boerlin *et al*, fig. 1C (included below for comparison). The cost terms are *μ* = 10^−6^ and *ν* = 10^−5^ and the voltage decay constant is *τ*_*v*_ = 20. The noise added to the voltage dynamics and the stimulus was Gaussian with *σ*_*v*_ = 10^−3^ and *σ*_*c*_ = 0.01, respectively. When enforcing the constraint that one neuron should spike per time bin, we selected the neuron with the highest voltage above threshold. Another option with similar results would be to select randomly between the ones above threshold.

As a reminder, *N* is the number of neurons in the network and *W* is the vector of read-out weights. For the local framework, *α* is the slope of the exponential nonlinearity, *F*_*max*_ is the saturation, and *F*_*min*_ is the minimum firing rate. For the population framework, *κ* is the time window over which the network minimizes the error. Finally, *A* is the dynamics matrix of the linear dynamical system, *τ* is the decay time constant of the filtered spike trains **r**, and *d* is the time delay.

A software implementation of Poisson BSNs, along with code to re-generate figures shown in the manuscript, is available at https://github.com/pillowlab/PoissonBalancedNets.

### Cost terms

The original BSN model objective function incorporated two additional cost terms to penalize spiking: a quadratic cost term *μ* and the linear cost term *ν*. These terms encouraged the network to use fewer spikes and to distribute spiking more evenly across neurons with large and small output weights. We did not incorporate these in our derivation for clarity, but they are included in our simulations of the BSN.

Including both cost terms into the derivation in the Results section, Eq (4) becomes

$$\begin{array}{cc} E_{t} & =\left|\right|x_{t}-{\hat{x}}_{t}{\left|\right|}_{2}^{2}+\nu ||r_{t}{\left|\right|}_{1}+\mu ||r_{t}{\left|\right|}_{2}^{2} \end{array}$$ (33)

and the voltage and threshold equations become

$$\begin{array}{cc} v_{t} & =W{}^{⊤}\left(x_{t}-{\hat{x}}_{t}\right)-\frac{\mu }{\tau }r_{t} \end{array}$$ (34)

$$\begin{array}{cc} {\dot{v}}_{t} & =-\frac{1}{\tau_{v}}v_{t-1}+\Omega r_{t}-\left(W{}^{⊤}W+\frac{\mu }{\tau^{2}}\right)s_{t}+W{}^{⊤}c_{t} \end{array}$$ (35)

$$\begin{array}{cc} T_{i} & =\frac{\frac{\nu }{\tau }+\frac{\mu }{\tau^{2}}+\left|\right|W_{i}{\left|\right|}^{2}}{2} \end{array}$$ (36)

The linear cost term (*ν*) is proportional to the L1 norm of **r**(*t*), or ${\left||r|\right|}_{1}=\sum_{i=1}^{N}r_{[i]t}$. This cost term penalizes the network’s total activity. The quadratic cost term (*μ*) limits individual neuron firing rates, forcing a spread of activity across all neurons in a population. Over time, the network transfers activity from precise, costly neurons with high firing rates to imprecise, larger weighted neurons to maintain a compromise between efficiency and accuracy of the read-out. Boerlin *et al* also include a voltage leak term for biological realism.

In our Poisson models, we did not observe the ping-pong effects described in Boerlin *et al* for the range of parameters we considered, so we don’t need cost terms for network stability. For the local Poisson framework, the cost terms can be included when *α* and *F*_*max*_ are high enough to cause ping-ponging.

### Performance metrics

We use *R*^2^ as a measure for how well the network read-out is approximating the target variable. The formula for calculating these is

$$\begin{array}{c} R^{2}=1-\frac{\sum_{t=0}^{T}{\left({\hat{x}}_{t}-x_{t}\right)}^{2}}{\sum_{t=0}^{T}{\left({\hat{x}}_{t}-\hat{\bar{x}}\right)}^{2}} \end{array}$$ (37)

for a simulation of time length *T*, where $\hat{\bar{x}}$ is the mean of $\hat{x}$ over the entire simulation. The root-mean-squared error (RMSE) is simply

$$\begin{array}{c} RMSE=\frac{1}{T}\sum_{t=0}^{T}{\left({\hat{x}}_{t}-x_{t}\right)}^{2} \end{array}$$ (38)

The cross- and auto-correlations between spike trains were calculated as unbiased estimates $\hat{r}$ with a maximum lag of *l* = 50 time bins according to

$$\begin{array}{c} \hat{r}=\frac{1}{T-l}\left(\sum_{n=0}^{T-l-1}x_{n}y_{n+l}\right) \end{array}$$ (39)

where *x* and *y* are spike trains from two different neurons.
